# Factors Contributing to the Evolution of *mecA*-Mediated β-lactam Resistance in Staphylococci: Update and New Insights From Whole Genome Sequencing (WGS)

**DOI:** 10.3389/fmicb.2018.02723

**Published:** 2018-11-13

**Authors:** Maria Miragaia

**Affiliations:** Laboratory of Bacterial Evolution and Molecular Epidemiology, Instituto de Tecnologia Química e Biológica António Xavier, Universidade Nova de Lisboa, Oeiras, Portugal

**Keywords:** β-lactams resistance, *Staphylococcus sciuri*, staphylococcal cassette chromosome *mec* (SCC*mec*), methicillin-resistant *Staphylococcus aureus* (MRSA), whole genome sequencing

## Abstract

The understanding of the mechanisms of antibiotic resistance development are fundamental to alert and preview beforehand, the large scale dissemination of resistance to antibiotics, enabling the design of strategies to prevent its spread. The *mecA*-mediated methicillin resistance conferring resistance to broad-spectrum β-lactams is globally spread in staphylococci including hospitals, farms and community environments, turning ineffective the most widely used and efficient class of antibiotics to treat staphylococcal infections. The use of whole genome sequencing (WGS) technologies at a bacterial population level has provided a considerable progress in the identification of key steps that led to *mecA*-mediated β-lactam resistance development and dissemination. Data obtained from multiple studies indicated that *mecA* developed from a harmless core gene (*mecA1*) encoding the penicillin-binding protein D (PbpD) from staphylococcal species of animal origin (*S. sciuri* group) due to extensive β-lactams use in human created environments. Emergence of the resistance determinant involved distortion of PbpD active site, increase in *mecA1* expression, addition of regulators (*mecR1, mecI*) and integration into a mobile genetic element (SCC*mec*). SCC*mec* was then transferred into species of coagulase-negative staphylococci (CoNS) that are able to colonize both animals and humans and subsequently transferred to *S. aureus* of human origin. Adaptation of *S. aureus* to the exogenously acquired SCC*mec* involved, deletion and mutation of genes implicated in general metabolism (auxiliary genes) and general stress response and the adjustment of metabolic networks, what was accompanied by an increase in β-lactams minimal inhibitory concentration and the transition from a heterogeneous to homogeneous resistance profile. Nowadays, methicillin-resistant *S. aureus* (MRSA) carrying SCC*mec* constitutes one of the most important worldwide pandemics. The stages of development of *mecA*-mediated β-lactam resistance described here may serve as a model for previewing and preventing the emergence of resistance to other classes of antibiotics.

## Introduction

Antimicrobial resistance threatens the effective prevention and treatment strategies of an increasing range of bacterial infections. In 21st century we are facing the real possibility that minor injuries and common infections can lead to death. A detailed understanding of the evolutionary processes occurring in nature that lead to resistance development is thus essential for anticipating its emergence and to restrain its spread.

One of the best models of resistance development is the emergence of methicillin resistance in staphylococci not only due the fact that it is extremely well documented, but mainly because it gave rise to methicillin-resistant *Staphylococcus aureus* (MRSA) pandemics – presently a major public health concern (Oliveira et al., 2001, 2002; Lindsay, 2013; Otto, 2013).

Due to their high efficacy and low toxicity, β-lactams are the most widely used class of antibiotics (Shahid et al., 2009). They inhibit bacterial cell wall biosynthesis through irreversible binding to the traspeptidase domain of penicillin-binding proteins (PBPs) (Ghuysen, 1991, 1994).

*Staphylococcus* species have a broad distribution in nature and consist of large populations. They are common commensals of the skin and mucous membranes of humans and animals (Kloos, 1980, 1986, 1997) and are ubiquitously recovered from the environment (Huijbers et al., 2015). Although during most of its existence they live as mere colonizers, when the skin and mucous membranes barrier of their host is impaired and the host is immunocompromized staphylococci may arise as important pathogens. Among all staphylococcal species, *S. aureus*, is considered to be the most pathogenic, being associated to a myriad of infections ranging from mild skin infections to life-threatening diseases (Crossley and Archer, 1997).

The major driving force for the emergence of β-lactams resistance in staphylococci was the continuous exposure to β-lactams in multiple environments: in soils where they had to co-exist with penicillin-producing fungi; in production animal farms wherein large amounts of β-lactam antibiotics were used as food additives (National Research Council, 1980; Castanon, 2007), and during treatment of bacterial infections (Westh et al., 2004).

All MRSA contain a copy of an exogenous *mec* gene that codifies for PBPs with low affinity for β-lactams (*mecA, mecB, mecC*, and *mecD*) (Hartman and Tomasz, 1984; Harrison et al., 2013; Gomez-Sanz et al., 2015; Schwendener et al., 2017; Becker et al., 2018; Schwendener and Perreten, 2018), In this review we will focus on the evolution and emergence of methicillin resistance mediated by *mecA* which encodes an extra PBP, PBP2A (Hartman and Tomasz, 1984) with a low binding affinity to virtually all β-lactams. In the presence of β-lactams antibiotics the transpeptidase domain of all native PBPs is inactivated, but bacteria containing *mecA* continue to synthesize cell wall as a result of the cooperation between transpeptidase domain of the PBP2A and the transglycosylase domain of the native staphylococcal PBP2 (Pinho et al., 2001). The few β-lactam to which *mecA* does not confer resistance include ceftobiprole and ceftaroline which are active against MRSA (Entenza et al., 2002; Ishikawa et al., 2003) and penicillin G, ampicillin and amoxicillin which are active against penicillinase-negative MRSA strains (a minority, nowadays).

Several efforts have been made to clarify the origin of *mecA*-mediated resistance to β-lactams in staphylococci and the use of state-of-the-art WGS technology has provided unprecedented advances (see Table 1). Nevertheless, the precise steps that led to β-lactam resistance development and dissemination are still not totally clear and are a matter of speculation.

**Table 1:** Insights into β-lactam resistance development provided by WGS.

***mecA* evolution**
• *mecA* homologs are ubiquitous in *S. fleurettii* and *S. vitulinus*
•*mecA* homologue is present in the native location In *S*. *fleurettii* and *S. vitulinus*
• *mecA* and *mecA2* in *S*. *vitulinus* do not provide resistance to β-lactams
•*mecA* homologs diversification begun with the use of antibiotics (1940s)
•*rnecB* and *mecC* were Identified
***SCCmec* evolution**
•*mec* complex and SCC elements evolved in parallel in different chromosomal locations
• *mecA* homologs native location in *S*. *sciuri* species group is 200 Kb from *orfX*
•*mecA and mecR2* originated from *S. sciuri* homologs
• *mecRl/mecl* were added to *mecA* to form the *mec* complex in *S. fleurettii* or *S. vitulinus*
• The last donors of J1 region and *ccr* to SCC*mec* were *S. sciuri*
• The last donors of *mec* complex, J2 and J3 regions to SCC*mec* were *S. vitulinus/S. fleurettii*
• Origin of SCC and SCC*rnec* is probably *S. sciuri*
•*mecA* was probably integrated into a SCC in 5. *sciuri*
• SCC*mec* III was probably the most ancient SCC*mec* type
**Expression of β-lactam resistance**
• Genetic basis of hetero-to-homo resistance conversion
– Tandem amplification of SCC*mec*
– Mutations in *relA* and *rpoB*
• Mechanisms of β-lactam resistance development in *S. sciuri* and S. *vitulinus*
– Alterations in *mecAl/mecA2* promoter
– Alterations in PbpD structure
– SCC*mec* acquisition

## History of β-Lactams and β-Lactam Resistance

Penicillin, a natural antibacterial compound produced by fungi, was first discovered in 1928 by Alexander Fleming (Fleming, 1929). However, due to low production yield, instability of the compound and problems in purification it was only later, in 1941, that penicillin was used as an antibiotic to treat human bacterial infections. The necessity to treat sick and wounded soldiers in the Second World War promoted the mass production of penicillin, and in 1945 this antibiotic was already used routinely in human clinical practice (Aminov, 2017).

Based on studies showing that penicillin is a growth promoter of chickens, pigs and livestock (National Research Council, 1980; Castanon, 2007), in 1951, the Food and Drug Administration (FDA) also approved the use of penicillin in animals (Hao et al., 2014). Nowadays, penicillin and other β-lactams continue to be used in many countries in food production animals not only to enhance animal growth, but also to treat infections and as a prophylactic (Hao et al., 2014). In fact, recent surveillance studies in Europe indicate that 25% of all antibiotic consumption (in mg/PCU) in veterinary setting relate to penicillin (report SE, 2010/2015), much of which are used for non-therapeutic purposes in chickens, cattle, and swine, compared with just a small quantity used for clinical treatments (World Organization for Animal Health, 2016).

A natural consequence of penicillin exposure was the development of antibiotic resistance. In fact, in 1942, only 2 years after the introduction of penicillin into clinical practice, the first penicillin-resistant *S. aureus* emerged in a hospital (Barber and Rozwadowska-Dowzenko, 1948) and shortly after (1960s) were also disseminated in the community (Rountree and Freeman, 1955), reaching around 80%. Penicillin resistance emerged due to the acquisition of β-lactamases that were able to hydrolyze and inactivate penicillin. Further developments to overcome resistance to penicillin included the synthesis of penicillinase-resistant penicillins, such as methicillin in 1960. However, due to its amazing adaptative power, *S. aureus* that were resistant to methicillin and to all β-lactams emerged right after its first use in the treatment of bacterial infections, through the acquisition of *mecA* (Jevons, 1961). This event has lead to the emergence of methicillin-resistant *S. aureus* (MRSA) strains and to one of the most important bacterial pandemics in hospitals worldwide (Oliveira et al., 2002; Grundmann et al., 2006). The MRSA rates in hospitals increased then, exponentially, reaching extremely high levels (above 60%) in the 1990s, mainly in Southern European countries (Deurenberg and Stobberingh, 2008; Chambers and Deleo, 2009; Lindsay, 2013). Similar to what was observed before for penicillin, this was followed by a wave of MRSA emergence in the community (Herold et al., 1998; Continuous Discharge Certificate [CDC], 1999; Vandenesch et al., 2003; Vlack et al., 2006), causing infections in otherwise healthy persons. Community-associated MRSA (CA-MRSA) are nowadays endemic in the community in specific countries like United States and here they have also become major multidrug resistant hospital clones (Moran et al., 2006; Otto, 2013). Additionally, resistance to β-lactams has expanded into farm environments wherein specific MRSA clones, like ST398 have become frequent colonizers of production animals and of humans in contact with them (Armand-Lefevre et al., 2005; Voss et al., 2005; Fluit, 2012).

As was observed for β-lactams, the emergence of drug resistance has been described following the introduction of each new antimicrobial class. The recent awareness by political authorities of the problem of antimicrobial resistance has lead to actions toward the banning of antimicrobial use in animals (European Union [EU], 2003; Food and Drug Administration [FDA], 2018). However, rules controlling antimicrobial use in animals have been applied mainly in Europe (National Research Council, 1980; World Organization for Animal Health, 2016), and still some of the antimicrobials, like penicillins, used to treat human disease continue to be heavily used in animals in European countries (Grave et al., 2012).

## The Structural Element of β-Lactam Resistance: the *mecA* Gene

β-lactams target the PBPs, involved in the synthesis of peptidoglycan, the major structural component of the bacterial cell wall. In particular, PBPs catalyze the main reactions involved in the polymerization of peptidoglycan, namely the elongation of glycan strands (transglycosilation) and the cross-linking between stem peptides of different glycan strands (transpeptidation) (Macheboeuf et al., 2006). Binding of β-lactams to native PBPs turns them inactive, what prevents peptidoglycan synthesis and bacterial growth (Waxman and Strominger, 1983). This reaction involves the break of the β-lactam ring amide bond and acylation of the PBPs, which gives rise to a serine ester-linked acyl derivative that is extremely stable and has a low rate of deacylation.

The *mecA* gene encodes a high-molecular weight class B PBP, called PBP2A (Hartman and Tomasz, 1984), which contains two domains, the C-terminal domain which is known to have a transpeptidation function, and a N-terminal domain to which no function has been attributed, the so called non-binding (NB) domain. Resistance is provided by the fact that this extra PBP has a lower efficiency of acylation by β-lactams, which is believed to result from a lower affinity for these compounds (Fuda et al., 2004) and from a slow rate of acylation. Resolution of PBP2A crystal structure showed that the poor acylation rate observed is due to the presence of a distorted active site, provided by a higher flexibility of the NB domain and by differences in regions close to the active site groove in the transpeptidase domain (Lim and Strynadka, 2002). Furthermore, the position of Ser403 was considered crucial for the effective nucleophilic attack of the β-lactam ring, which leads to acylation of the protein (Lim and Strynadka, 2002).

## The Mobile Element Carrying *mecA*: SCC*mec*

**FIGURE 1:**
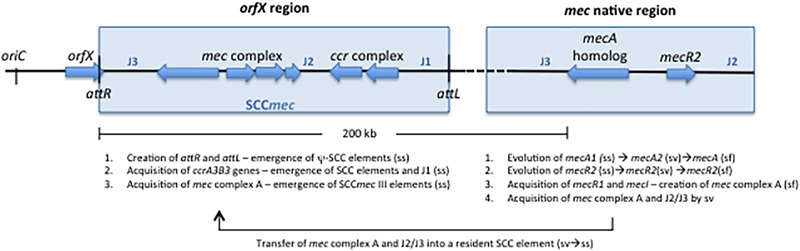
Schematic representation of the genomic events possibly associated with the evolution and assembly of SCC*mec* III occurring in the *orfX* and *mec* native region of species of *Staphylococcus sciuri* group. The basic structure of SCC*mec* in *orfX* is shown, including *attL, attR, mec* complex, *ccr* complex, J1, J2, and J3 regions. The elements present in the native region in species of the *S. sciuri* group (*S. sciuri, S. vitulinus*, and *S. fleurettii*) are shown, including a *mecA* homolog, the *mecR2*, J2 and J3 regions. *S. sciuri*: ss; *S. vitulinus*: sv; *S. fleurettii*: sf.

The *mecA* gene is carried in a mobile genetic element called staphylococcal cassette chromosome *mec* (SCC*mec*) (International Working Group on the Classification of Staphylococcal Cassette Chromosome Elements [IWG-SCC] et al., 2009). SCC*mec* is delimited by distinctive terminal inverted and direct repeats formed upon SCC*mec* insertion (DR-left downstream *orfX*, attL; DR-right at SCC*mec* end, attR) in a single chromosomal location, in the 3′ end of *orfX*, a RNA methyltransferase that is localized near the origin of replication (Boundy et al., 2013). This mobile genetic element is composed of two central elements, the *mec* complex containing *mecA* and intact and deleted forms of its regulators (*mecI, mecR1*) and the *ccr* complex composed of cassette chromosme recombinases (*ccr*) involved in its mobility (Katayama et al., 2000). The remaining portions of SCC*mec*, are composed of non-essential components, namely additional metal and antibiotic resistance genes carried by transposons and plasmids, as well as genes of unknown function, which are named J regions. The J3 region is located between *orfX* and *mec* complex, the J2 region is flanked by *mec* complex and the *ccr* complex and the J1 region between *ccr* complex and the right extremity of the element (see Figure 1). More recently, *mecR2*, coding for an anti-repressor of *mecA* was described to exist downstream *mecI*, which together with *mecI* and *mecR1* constitute an unusual three-component arrangement (Arede et al., 2012). So far as many as thirteen different structural types of SCC*mec* have been described in *S. aureus*^1^ (Ito et al., 2001, 2004; Ma et al., 2002; Oliveira et al., 2006; Berglund et al., 2008; Zhang et al., 2009; Garcia-Alvarez et al., 2011; Li et al., 2011; Wu et al., 2015; Baig et al., 2018) that range between 20 and 70 Kb. The different types of SCC*mec* correspond to different combinations of *mec* complex class (A-E), according to the presence/absence of regulatory genes and insertion sequences, and *ccr* allotypes (*ccrAB* and *ccrC*).

SCC*mec* is believed to have been acquired a limited number of times in *S. aureus* (Robinson and Enright, 2003), but acquisition of this element appears to provide a major advantage to bacteria mainly in the hospital environment. In fact, the acquisition of different types of SCC*mec* by methicillin-susceptible *S. aureus* (MSSA) of diverse genetic backgrounds gave rise to several MRSA pandemics over time (Chambers and Deleo, 2009), namely the Iberian (ST247-I), Brazilian (ST239-III), New York-Japan (ST5-II), EMRSA16 (ST36-II), EMRSA15 (ST22-IV), Berlin (ST45-IV), United States 300 (ST8-IV), and ST398-V (Deurenberg and Stobberingh, 2009). In contrast to *S. aureus*, in coagulase-negative staphylococci (CoNS), there is not a clear association between SCC*mec* and specific genetic backgrounds (Rolo et al., 2012). However, it is still not clear if this derives from the fact that SCC*mec* was acquired a higher number of times or from a higher instability of SCC*mec* structure in these species. Nevertheless, specific SCC*mec* types appear to be more common to certain CoNS species. SCC*mec* type I and VI are more common in *Staphylococcus hominis* (Bouchami et al., 2012), SCC*mec* III in *Staphylococcus sciuri* group of species (Rolo et al., 2017a), SCC*mec* IV in *S. epidermidis* (Miragaia et al., 2007), and SCC*mec* V in *S. haemolyticus* (Bouchami et al., 2010).

The true breadth of genetic diversity of SCC*mec* elements is still unknown. While in *S. aureus* this structure has been shown to be relatively stable, in CoNS, such as *S. epidermidis* and *S. haemolyticus*, a high genetic diversity has been described (Miragaia et al., 2007). This might be due to the fact that CoNS species have a high recombination rate (Miragaia et al., 2007), an enhanced ability to acquire and maintain exogenous genetic material or because these elements have been acquired earlier by these species than by *S. aureus*. In the era of WGS where detailed information on the entire genomes of thousands of staphylococci are being gathered, the number of types and subtypes of SCC*mec* have increased exponentially^1^, and this challenges the traditional criteria and methodologies that were previously defined to classify SCC*mec* types, mainly based on PCR. A web-tool, SCC*mec*Finder, able to identify all SCC*mec* element types (I to XIII) and SCC*mec* IV and V subtypes was recently developed to classify SCC*mec* types based on WGS data (Kaya et al., 2018). The characterization of the SCC*mec* elements is based on two different gene prediction approaches to achieve correct annotation.

## The SCC Elements

Although due to its clinical relevance, SCC*mec* is the most popular element, several other SCC elements not carrying the *mec* complex (SCC) or either *mec* complex and *ccr* complex (pseudo-SCC) have been identified at the *orfX* site. These elements can carry diverse genes relevant for staphylococcal survival and virulence, namely conferring heavy metal resistance genes (Chongtrakool et al., 2006) providing capsule production (Luong et al., 2002), cell-wall biosynthesis (Mongkolrattanothai et al., 2004), restriction/modification functions or immune protection (Holden et al., 2004). These elements can be found alone in the chromosome or in tandem with SCC*mec* or other SCC elements, being in this case named composite islands (CI). Examples of such CIs include the SCC*mec* III-SCC*mer* from the pandemic MRSA Brazilian clone (Chongtrakool et al., 2006) and the SCC*mec* IV-ACME from the USA300 CA-MRSA clone (Shore et al., 2011b). Although many studies have described the structure and contents of these elements, few studies have addressed their true contribution for staphylococcal virulence or fitness (Diep et al., 2008).

Besides being inserted at the exact same location as SCC*mec*, SCC and pseudo-SCC elements have been described to have regions of homology with SCC*mec* (Katayama et al., 2003a) (see Table 2), suggesting that their evolutionary history is related. Still, until recently the nature of their relatedness remained elusive.

**Table 2:** Nucleotide identity (%) of SCC elements found *in S. sciuri, S*. *vitulinus* and *S*. *fleurettii* with S. *aureus SCCmec.*

SCC element	Species	*ccr* type	*mec* complex	Homology with *S. aureus* SCC*mec* region (%)
SCC_11/01_	S. *sciuri*	*ccrA5B5*	–	J1: IVa (46), SCC*mec* S. *xylosus (mecC)* (59)
				J2: III (67–96), IX (79–93)
				J3: IX (69–91)
SCC_K6-15937_	*S. sciuri*	*ccrA5Bnew*	–	J1:III(90), V(79)
				J2: III (95)
				J3: IX (99)
ψ-SCC_K6-10930_	*S. sciuri*		–	Jl: V (65)
				J3: III (55–80)
SCC*mec* III-like B	*S. sciuri*	*ccrA3B5*	A	J2: II (99–100), III (60–100). IX (73) J3: III (100)
SCC*mec* III-like A	*S. sciuri*	*ccrA3B5*	A	J1: X (96), III (90–100)
				J2: III (38–100)
				J3: IV (42)
SCC_K116_	*S. sciuri*	*ccrC*	–	J2: II (90), III (91–98), V (73–80)
SCC-CI_SS27_-I SCC-CI_SS27_ - II	*S. sciuri*	*ccrA1B3 ccrA1B3*		J2: III(77), X(90)
				Jl: IX (77), XI (73)
				J2: II (94), IV (62–79), IX (73–97)
SCC_402567_	*S. fleuretti*	*ccrAnewB5*	–	J2: IX (75), III (77–96)
SCC_11683_	*S. vitulinus*	*ccrABnew*	–	J1: IX (56–95) J2: IX (97)
SCC_401946_	*S. vitulinus*	*ccrABnew*	–	J1: IX (76), V (82) J3: IV (51)

## Transfer of SCC Elements

SCC*mec* with exactly the same nucleotide sequence were found in different staphylococcal strains and species, suggesting that this element is frequently transferred among staphylococci (Shore et al., 2011b). However, the mechanisms of SCC and SCC*mec* insertion and excision from the chromosome as well as the mechanism of transfer is still elusive and it is likely that several different mechanisms are involved.

When grown in antibiotic free medium SCC*mec* can be excised from the chromosome, a reaction catalyzed by the cassette chromosome recombinases (Ccr) (Katayama et al., 2000). Immediately after excision, SCC*mec* circularizes into an extrachromosomal intermediate form and the attSCC sites are created in the chromosome (*attB*) and the intermediate circular form (*attS*). The created attSCC sites, work like recognition sites for the Ccr enzymes for a later integration into the chromosome. During SCC*mec* excision and insertion *orfX* remains always intact (Katayama et al., 2000).

Electrophoretic mobility shift assay (EMSA) showed that Ccr enzymes recognize a minimum of 14-bp sequence in the terminal sequence of the *orfX* that contains always the conserved sequence (TATCATAA), which is also found in SCC*mec* (*attS*). However, DNA sequences flanking the *att* sites were shown to also have a role on the frequency and efficiency of SCC*mec* insertion (Wang et al., 2012). This sequence specificity was thus probably important in the epidemiology of SCC*mec* acquisition by staphylococci and might partly explain the association of some SCC*mec* to specific genetic backgrounds or staphylococcal species (Oliveira et al., 2002; Miragaia et al., 2007; Bouchami et al., 2010, 2012; Rolo et al., 2017a).

According to studies of translational fusions of the *ccr* promoter with green fluorescent protein the *ccr* activity and associated SCC*mec* excision is a bistable process occurring only in a small fraction of cells within the population (Stojanov et al., 2013). The fate of the extrachromosomal circularized SCC elements after they are formed is still a mystery. SCC elements have once been regarded as non-replicative, due to the absence of a replication origin, but recent crystallographic studies have provided evidence that SCC*mec* elements encode an active MCM-like helicase (Mir-Sanchis et al., 2016), suggesting that they can eventually replicate in the cytoplasm before being transferred, but further studies are needed to confirm these observations.

The transfer of SCC*mec* have been successfully achieved in the laboratory by several different genetic mechanisms. In particular susceptible strains in contact with phage lysates containing this mobile genetic element were shown to become resistant to β-lactams and SCC*mec* elements were successfully packaged into bacteriophage capsides (Cohen and Sweeney, 1970; Maslanova et al., 2013; Scharn et al., 2013; Chlebowicz et al., 2014; Haaber et al., 2016). Additionally, transfer of SCC*mec* was also achieved through natural transformation, upon induction of SigH in very specific laboratory growth conditions (Morikawa et al., 2012). Moreover, a chromosomally encoded and laboratory-constructed derivative of SCC*mec* was captured on a conjugative plasmid and transferred by filter-mating into different *S. aureus* and *S. epidermidis* recipients (Ray et al., 2016). Also, evidence of possible transfer of SCC*mec* by conjugation was the finding of a *mecA* homologue within a plasmid of *Macrococcus caseolyticus*, a species phylogenetically related to *Staphylococcus* (Tsubakishita et al., 2010a).

In spite of the huge effort of the scientific community in elucidating the mechanism of SCC*mec* transfer, many of the studies described occurred in very artificial conditions and it remains to be clarified which mechanism(s) are actually more frequent *in vivo*.

## The Origin of the Methicillin Resistance Determinant – *mecA* Evolution in *S. sciuri* Group

Early studies, based on structural nucleotide identity, have proposed that the *mecA* gene originated from recombination between a PBP from *E. coli* (Song et al., 1987) or from *Enterococcus hirae* (Archer and Niemeyer, 1994) with a β-lactamase encoding gene. A theory that was later supported by the finding by WGS of another *mec* allotype (*mecC*) as part of a class E *mec* complex, containing *blaZ* (*mecI-mecR1-mecC-blaZ*) in *Macrococcus caseolyticus* (Tsubakishita et al., 2010a) and *S. xylosus* (Harrison et al., 2013).

Other lines of evidence suggest that *mecA* originated from native PBPs of species of the *Staphylococcus sciuri* group – a primordial phylogenetic clade, including *S. sciuri, Staphylococcus fleurettii, Staphylococcus vitulinus, Staphylococcus lentus*, and *Staphylococcus stepanovicci* (Schleifer et al., 1983; Couto et al., 1996; Zhou et al., 2008; Antignac and Tomasz, 2009; Hauschild et al., 2010; Tsubakishita et al., 2010b), which most important ecological niches are the soil and skin and mucous membranes of wild and production animals.

**FIGURE 2:**
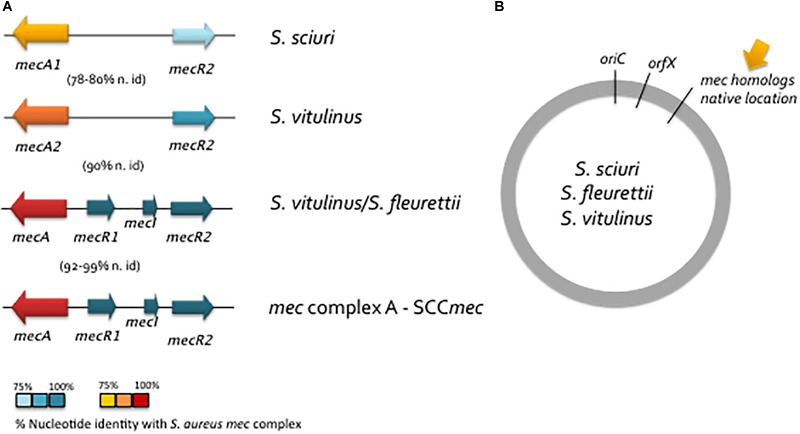
Schematic representation of the structure of *mec* native region in *S. sciuri* group species. **(A)** Structure of *mec* native region in *S. sciuri, S. vitulinus* and *S. fleurettii* in comparison to *mec* complex A in *S. aureus*. Colors indicate the level of identity of the *mec* complex A from *S. aureus* with the corresponding region in *S. sciuri* species group as depicted in Figure legend. **(B)** Location of the *mec* native region in the *S. sciuri* group of species.

**FIGURE 3:**
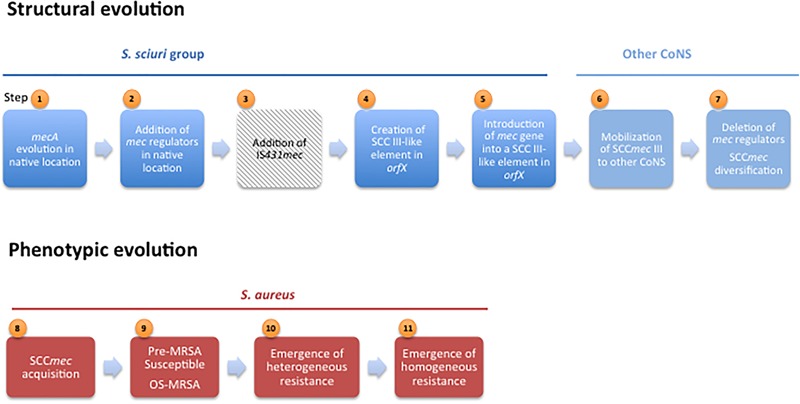
Steps in structural and phenotypic evolution of *mecA*-mediated β-lactam resistance.

The use of WGS on a large collection of isolates belonging to the *S. sciuri* group revealed the presence of homologues of *S. aureus mecA* with different levels of homology that were ubiquitous within some of the species (Couto et al., 1996; Wu et al., 1996; Hiramatsu et al., 2013) (see Figure 2A). However, in contrast to *mecA* in *S. aureus, mecA* homologs in *S. sciuri* group species were all located approximately 200 Kb apart from *orfX* (native location) between *mva* and *xyl* operons, outside any SCC element (Rolo et al., 2017b) (see Figure 2B). The results suggest that *mecA* has been transmitted vertically during the early stages of staphylococcal speciation (see Figure 2A; Step 1, Figure 3).

Although the primary function of these *mecA* precursors was probably related to cell wall synthesis and not to antimicrobial resistance, the complete evolution from a native PBP into a resistance determinant appears to have been a stepwise process that occurred within this group of species. *S. sciuri* carries the most ancestral form of *mecA* (*mecA1*) which has 85% homology in nucleotide sequence with *S. aureus mecA*; *S. vitulinus* harbors an intermediary form (*mecA2*) with 94% homology and all *S. fleurettii* and some *S. vitulinus* have a *mecA* form that is almost identical to that of *S. aureus mecA* (*mecAf, mecAv*; 99% homology) (Rolo et al., 2017b; Tsubakishita et al., 2010b) (see Figure 2 and Table 3). In *S. lentus* and *S. stepanovicii* so far no *mecA* homolog has been described, (Tsubakishita et al., 2010b; Calazans-Silva et al., 2014), but no extensive and detailed study was ever performed in these two species.

**Table 3:** Nucleotide identity (%) of chromosoomal regions of *S. sciuri, S. fleurettii* and *S. vitulinus* with those found within *S. aureus* SCC*mec*.

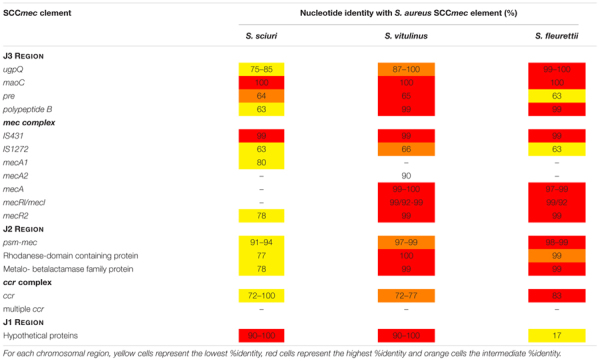

The only *mecA* homologue that confers resistance to β-lactams is the *mecA* in *S. fleuretti*. The same *mecA* homologue in *S. vitulinus* and the *mecA1* and *mecA2* found in *S. sciuri* and *S. vitulinus*, respectively, do not confer resistance to β-lactams in the great majority of strains (Couto et al., 1996; Wu et al., 1998; Monecke et al., 2012; Rolo et al., 2017b). However, *S. sciuri* and *S. vitulinus* strains exhibiting β-lactam resistance have been reported (Couto et al., 2003; Tsubakishita et al., 2010b; Rolo et al., 2017b). Recent work by Rolo et al. (2017b) wherein a large collection of *S. sciuri* and *S. vitulinus* were analyzed by WGS showed that β-lactam resistance in these species emerged multiple times during evolution and was driven mainly by the contact with human created environments, namely with the beginning of the use of antibiotics in production animals and humans. The mechanisms of resistance development in these two species included: (i) the structural diversification of the non-binding domain of native PBPs which altered the structure of the active site and exposure of ser403; (ii) mutations and insertion of IS*256* in the promoters of *mecA* homologs that were associated to an increased expression of the proteins; (iii) acquisition of SCC*mec.* Additionally, like in *S. aureus*, the bacterial genetic background plays an important role in the expression of β-lactam resistance in the *S. sciuri* group of species, since the exact same gene allele was found associated to both susceptible and resistant strains (Rolo et al., 2017b).

Additional evidence supporting that *S. sciuri mecA1* was the evolutionary precursor of *S. aureus mecA*, include the fact that this gene could be recruited to express methicillin resistance in *S. sciuri* after stepwise exposure to methicillin (Wu et al., 2001). Moreover, the activated copy of *S. sciuri mecA1* was, similarly, able to restore methicillin resistance phenotype, when transduced into methicillin-susceptible *S. aureus* (MSSA), conferring high level, homogeneous and broad-spectrum β-lactam resistance (Wu et al., 2001). Furthermore, the *S. sciuri mecA1* when transduced into MSSA was shown to act exactly like *S. aureus mecA*, being controlled by *S. aureus mecA* regulators (*mecI* and *mecR1*) and its product (PBP4) taking part in cell wall biosynthesis, producing a peptidoglycan typical of methicillin-resistant *S. aureus* (Antignac and Tomasz, 2009).

Besides *mecA*, other *mec* genes have been identified that are associated with β-lactam resistance, namely *mecB* and *mecD* in *Macrococcus caseolyticus* (Baba et al., 2009; Schwendener et al., 2017; Schwendener and Perreten, 2018) and *mecC* in *S. aureus* (Garcia-Alvarez et al., 2011; Shore et al., 2011a), *S. xylosus* (Harrison et al., 2013), *S. sciuri carnaticus* (Harrison et al., 2014), and *S. stepanovicii* (Loncaric et al., 2013). The *mecB* and *mecD* are the most distant from *S. aureus mecA*, having, respectively, a nucleotide identity with *mecA* that is equal or lower than 62%, whereas *mecC* has 69% nucleotide sequence identity. All *mec* forms confer resistance to β-lactams to their natural hosts and their introduction into a susceptible *S. aureus* genetic background was able to provide a resistance phenotype, confirming that they should encode a PBP with low-affinity to β-lactams that participates in cell wall synthesis (Baba et al., 2009; Kim et al., 2012). Both *mecB* and *mecC* were carried within mobile genetic elements structurally similar to SCC*mec* that were inserted in the *orfX* region (SCC*mec* XI, SCC*mec* IX-like) (Gomez-Sanz et al., 2015) and *mecB* was additionally found within a plasmid in *M. caseolyticus* (Baba et al., 2009; Tsubakishita et al., 2010a). The *mecD* gene is carried within a resistance island (McRI*_mecD_*-1, McRI*_mecD_*-2) that is inserted in 3′ end of the *rpsI* gene. Besides *mecD* this island contains genes for an integrase of the tyrosine recombinase family, but does not resemble either SCC elements or *mecB*-carrying mobile genetic elements (Schwendener et al., 2017; Schwendener and Perreten, 2018). However, none of the *mecB, mecC* or *mecD* was found within the native location (200 Kb apart from *orfX*).

The exact evolutionary link between *mecA, mecB, mecC* and *mecD* forms is still undetermined. Among all *mec* genes, *mecA* is apparently, the most successful in *Staphylococcus*. The *mecB* was recently found within a plasmid in a single *S. aureus* human carriage strain belonging to ST7 (Becker et al., 2018) and *mecC* has been limited to only a few *S. aureus* clonal lineages (CC130 and ST425) and four *Staphylococcus* species (*S. sciuri, S. xylosus, S. stepanovicci*, and *S. aureus*) (Harrison et al., 2013, 2014; Loncaric et al., 2013; Becker et al., 2014; Semmler et al., 2016). MRSA harboring *mecC* are believed to have a zoonotic origin and although they were reported in several different countries, they have been rarely observed in human infection (Becker et al., 2014). However, surveillance of dissemination of these *mec* genes should not be disregarded, since antibiotic use and the consequent selective pressure could drive fast evolutionary leaps that can lead to their precipitous spread.

## Stages in the Evolution of SCC*mec*

Most of the efforts have been focused on the clarification of the origin and evolution of the β-lactam resistance determinant (*mecA*). Much less information is available regarding the evolution of SCC*mec*, the mobile element carrying *mecA*, which is responsible for the worldwide spreading of β-lactam resistance among staphylococci. SCC*mec* is a mosaic-like element that was described to contain multiple transposable elements, plasmids and insertion sequences in J regions (Ito et al., 2001), a genetic environment that *per si* can promote and facilitate genetic variation and recombination, what has been hindering the reliable tracing of their phylogeny.

The characterization of the native location of *mecA* homologs, the SCC insertion site and the genetic background of a large collection of isolates belonging to *S. sciuri* group by comparative genomics showed that SCC elements and *mecA* and flanking regions evolved in parallel in these species in these two distinct chromosomal locations (Rolo et al., 2017b).

### Assembly of the *mec* Complex in the Native Location

The *mecA* homologs flanking genes in the native location were found to be the same as those flanking *mecA* inside SCC*mec*, encompassing the J2 and J3 regions (Rolo et al., 2017b) (see Table 3). Moreover, as for native *mecA* homologs, the level of homology of their flanking genes (*psm*-*mec* and *ugpQ*) with the same genes in SCC*mec* from MRSA, varied according to the phylogeny, wherein those of *S. fleuretti* were the most similar and those of *S. sciuri* were the most distant (Rolo et al., 2017b). The results suggest that the first stage of SCC*mec* evolution included the evolution of *mecA* homologs and their neighbor genes in the native location (Step 1, Figure 3). This was followed by the creation of the *mec* complex (Step 2, Figure 3). The *mecR2* was the first regulator to be added in *S. sciuri* at the native location near *mecA1*, since the most ancient precursor of *mecR2* was found in this species. This gene organization was preserved along phylogeny and became ubiquitous in *S. fleurettii* and *S. vitulinus* (Tsubakishita et al., 2010b). Addition of *mecR1* and *mecI* happened later, after the evolution of the ancestral *mecA1* into *mecA* was complete, as demonstrated by the lack of these regulators in *S. sciuri* and their occurrence in the native location of *S. fleurettii* and *S. vitulinus* near *mecA* (Tsubakishita et al., 2010b; Rolo et al., 2017b). These finding came to reconcile previous controversies, suggesting that although *mecA1* was the original precursor of *mecA, S. fleuretti*/*S. vitulinus* were probably the last donors of the *mec* complex to give rise to SCC*mec*. The addition of IS*431* element probably occurred later, after *mecA*, regulators and neighboring regions were mobilized into a SCC element located in the *orfX* region (Step 3, Figure 3). Alternatively, it could have been added during their mobilization, as it was never detected in the native location in any of the strains tested.

Acquisition and expression of *mecA* in species in which this gene is not native imposes a fitness cost to bacteria (Ender et al., 2004). For this reason, the step of addition of regulators with subsequent *mecA* repression appears to have been particularly crucial in the maintenance of the gene in new host species (Katayama et al., 2003b) and thus in *mecA* dissemination. In fact, some of the first methicillin resistant staphylococci, the so-called pre-MRSA (Step 9, Figure 3) (Hiramatsu et al., 1992; Kuwahara-Arai et al., 1996), contained intact regulators and a susceptible phenotype.

Studies wherein the *mec* complex region was characterized in MRSA and MR-CoNS revealed that although *mecI* and the 3′ end of *mecR1* are deleted in a great proportion of contemporary clinical strains (Katayama et al., 2001), the 5′ end portion of *mecR1* as well as a copy of IS*431* downstream *mecA* (IS*431*-R) are conserved in every strain. Moreover, deletion of *mecI* and *mecR1* promoted by IS*431* was accomplished *in vitro* upon selection with methicillin in a *S. haemolyticus* with a *mec* complex type A (Suzuki et al., 1993). These observations are in accordance with the view that the *mec* regulators and the IS*431*-R together with *mecA* were once the original components of the *mec* region DNA and that deletion of the regulators occurred at a later time in evolution (Step 7, Figure 3). On the other hand, the similarity of nucleotide sequence in regions located upstream of the *mec* complex in four different *mec* complex classes, suggests that deletion of the *mec* regulators must have occurred after the establishment of the prototypic *mec* complex A in a SCC*mec* element (Katayama et al., 2001).

Thus the assembly of *mec* complex A seems to be the first step of genetic evolution, followed by its establishment in SCC*mec* and subsequent deletion of the regulators to originate the different *mec* complex classes (*mec* complex B, C, D, E).

### SCC Element Evolution in the *orfX* Region

Analysis of the *S. sciuri orfX* region showed that SCC elements most probably originated in *S. sciuri* and were assembled from housekeeping genes located in this region (Step 4, Figure 3), as evidenced by the finding of the same housekeeping genes either outside or within SCC elements (Rolo et al., 2017a) – a finding not observed in the other species of the *S. sciuri* group. Additionally, in *S. sciuri* the most ancestral forms of cassette chromosome recombinases (*ccr*) and the highest genetic diversity were found, including almost all *ccr* allotypes described in *S. aureus* (Rolo et al., 2014). Interestingly, it was also in this species that a SCC*mec* type III-like was found with high homology simultaneously with the *mec* complex and J2 region of *S. aureus* SCC*mec* type III and the J1 region and an ancestral form of *ccrAB3* of *S. sciuri* SCC non-*mec* (Rolo et al., 2017a) (see Table 2). The results suggest that SCC*mec* III originated in *S. sciuri*, probably through the integration of the *mec* complex and J2 region from *S. vitulinus*/*S. fleuretti* into a resident SCC non-*mec* carrying *ccrAB3* (Step 5, Figure 3). However, the mechanism that mobilized the *mec* complex from *S. vitulinus*/*S. fleuretti* to an SCC in *S. sciuri* is still not known. Once formed SCC*mec* III probably disseminated to other CoNS species, namely *S. epidermidis* and *S. aureus* ST239, wherein SCC*mec* III was found to be prevalent (Miragaia et al., 2007; Harris et al., 2012) (Step 6, Figure 3).

Although it is apparent that the origin of SCC*mec* type III is *S. sciuri*, the source of the remaining SCC*mec* types elements remains unclear. In contrast to *S. sciuri*, which carried a high diversity of *ccr* allotypes, methicillin susceptible CoNS species belonging to more recent clades in the phylogeny of staphylococci, which include *Staphylococcus epidermidis, Staphylococcus haemolyticus* and *Staphylococcus hominis*, were particularly enriched in a specific allotype of *ccr*. The *ccrAB2* was found to be common in *S. epidermidis* (Miragaia et al., 2007), *ccrAB1* in *S. hominis* (Bouchami et al., 2012) and *ccrC* in *S. haemolyticus* (Bouchami et al., 2010), which is coincidently the same type of *ccr* carried by the most frequent SCC*mec* in these species. It is thus tempting to speculate that each SCC*mec* type can result from the integration of *mec* complex, probably through recombination, into a resident SCC element in these species. This hypothesis is supported by the identification of SCC non-*mec* elements carrying different *ccrAB* types in *S. aureus* and CoNS with regions of high homology with known SCC*mec* types (Katayama et al., 2003a). However, the enrichment of certain SCC*mec* types in particular species may also derive from the described specificity of the different types of Ccr enzymes as described above (Wang et al., 2012).

### SCC*mec* Diversification

The next stage of SCC*mec* evolution that is believed to be still ongoing includes the diversification and dissemination of the SCC*mec* element among the staphylococcal population (Step 7, Figure 3). The existence of similar regions among different SCC*mec* types, like the J1 region in SCC*mec* type II and IV or the *mec* complex B between SCC*mec* type I and IV (Chongtrakool et al., 2006) suggest that the different SCC*mec* types are related.

The involvement in the diversification process of species, such as *S. epidermidis, S. hominis* and *S. haemolyticus* is apparent. Besides being reservoirs of specific types of SCC*mec* and *ccr* allotypes, these species harbor a huge number of non-described SCC*mec* types (Wisplinghoff et al., 2003; Miragaia et al., 2007; Bouchami et al., 2010), evidencing their key role in the current diversification of SCC*mec*.

Moreover, factors associated to hospital environment appear to be driving the diversification and acquisition of SCC*mec* in species like *S. epidermidis* (Rolo et al., 2012). One of the factors related to the clinical setting that might be triggering SCC*mec* diversification is the use of antibiotics, namely β-lactams and vancomycin, which were already shown to promote the expression of recombinases (Higgins et al., 2009). The excision of SCC elements promoted by *ccr* overexpression may create new opportunities of recombination between different elements within the same strain, giving rise to new SCC*mec* structures.

## The Importance of Genetic Background for the Acquisition of SCC*mec* and for the Expression of β-Lactam Resistance in *S. aureus*

The transfer of SCC*mec* from CoNS to *S. aureus* was probably a subsequent step (Step 8). Several lines of evidence suggest that acquisition and expression of *mecA* by *S. aureus* was a complex process involving multiple genetic and metabolic alterations. The construction of a Tn*551* transposon library in the background of the MRSA strain COL and subsequent screening for a decreased level of methicillin resistance has identified several factors (fem or auxiliary genes) that, together with the *mecA* gene, are crucial for the expression of high-level and homogeneous resistance to methicillin (de Lencastre and Tomasz, 1994) (Step 11, see Figure 3). Although having a substantial impact on oxacillin resistance, these genes are not directly implicated in the expression of *mecA*, but they are mainly involved in cell wall metabolism and stress response (de Lencastre and Tomasz, 1994). However, these studies were performed in a single MRSA strain (COL) and these same genes appear to have different contributions to β-lactams resistance in other *S. aureus* genetic backgrounds (Memmi et al., 2008; Figueiredo et al., 2014), suggesting that expression of β-lactam resistance is extremely complex and that auxiliary genes in different MRSA strains might be different or use different mechanisms. Additionally, whether the identified auxiliary genes in COL also contribute to β-lactam resistance expression in other *Staphylococcus* species is unknown.

What appears to hold true is that not all *S. aureus* appear to have the same ability to accommodate *mecA*. The existence of a host barrier was evidenced by the finding that SCC*mec* was acquired by a limited number of *S. aureus* genetic backgrounds (e.g., ST239, ST45, ST22, ST8, ST5) (Robinson and Enright, 2003) while other genetic backgrounds despite being successful, like ST121, were rarely observed carrying *mecA* (Rao et al., 2015). Furthermore, when a recombinant plasmid, carrying intact *mecA*, was introduced into strains that have never experienced the presence of *mecA*, they were unable to maintain or express β-lactam resistance, a phenomena not observed when the same assay was performed in MSSA strains from which SCC*mec* has been excised (Katayama et al., 2003b). Interestingly, either the presence of β-lactamase (*blaR1*-*blaI*) or *mecA* regulatory genes (*mecR1*-*mecI*), which control *mecA* expression, allowed the maintenance and expression of plasmid-carried *mecA* in the naïve genetic background (Katayama et al., 2003b), which is indicative that besides the genetic backgrounds the repression of *mecA* was important for the acquisition and stability of *mecA* in staphylococci. Actually, the so-called pre-MRSA although carrying *mecA*, showed a susceptible phenotype, which was shown to result from *mecI*-mediated repression of *mecA* transcription (Kuwahara-Arai et al., 1996) (Step 9, Figure 3). The integration of newly acquired genes into the recipient metabolic network is a complex mechanism that frequently represents a large fitness cost for bacteria. The presence of the regulators will probably work as safeguard mechanisms that will silence the newly acquired gene and avoid potentially harmful consequences of its expression in the new bacterial host, while it is still not adapted (Ochman et al., 2000; Navarre et al., 2006).

A different phenomenon supporting the importance of genetic background for the expression of β-lactam resistance is the emergence of the so-called oxacillin susceptible MRSA (OS-MRSA) (Step 9, Figure 3), strains that like pre-MRSA carry *mecA* and do not express β-lactam resistance, but that in contrast do not carry *mecI* (SCC*mec* IV or V) (Giannouli et al., 2010; Andrade-Figueiredo and Leal-Balbino, 2016; Phaku et al., 2016). OS-MRSA have been recently described as a cause of infections in humans (Andrade-Figueiredo and Leal-Balbino, 2016) and have been also isolated from animals (Phaku et al., 2016). Functional and genomic analysis of OS-MRSA and MRSA strains identified mutations in *femA*, a known auxiliary gene, as the possible cause of the observed decreased resistance to β-lactams (Giannouli et al., 2010; Phaku et al., 2016). Although being described many years after the emergence of pre-MRSA, the exact date of OS-MRSA emergence is uncertain. Actually, since for several decades detection of MRSA in many hospitals was based in purely phenotypic approaches, OS-MRSA may have passed unnoticed. It could be that OS-MRSA correspond to strains that have recently acquired *mecA* and that have developed alternative mechanisms to compensate for the cost of acquisition of an exogenous gene.

Altogether, data suggest that for acquiring and maintaining *mecA, S. aureus* strains had to adapt its genetic background, compensated for *mecA*/SCC*mec* fitness cost, or were already intrinsically equipped for it. Still it remains to be clarified which genetic determinants and mechanism are involved in this adaptation process.

## Homogeneous and Heterogeneous Expression of Resistance to Methicillin

The genetic alterations in genetic backgrounds and associated metabolic alterations described above to have occurred upon SCC*mec* acquisition were frequently paralleled by alterations in the cell population profile of β-lactam resistance expression. Clinical MRSA isolates, when cultured, frequently exhibit a low level of methicillin resistance, but contain subpopulations of bacteria displaying very high levels of resistance to this antibiotic, a feature called heterogeneous resistance (Tomasz et al., 1991) (Step 10, Figure 3). Exposure of the hetero-MRSA strains to β-lactam antibiotics originates mutant strains in which all cells are uniformly highly resistant to β-lactams, named homogeneous methicillin resistance (Step 11, Figure 3) (Tomasz et al., 1991). Both hetero and homo resistance phenotypes can be found in clinical MRSA isolates, but they appear to correspond to two different and sequential evolutionary stages of β-lactams resistance expression. However, the molecular basis of the emergence of heterogeneous resistance and of the heterogeneous-to-homogeneous conversion is not totally understood and appears to derive by multiple different mechanism, of which only a few have yet been identified.

Genetic analysis of colonies within the highly resistant subpopulation of a heteregeneous MRSA strains, showed that high resistance was associated to the deletion of *lytH*, encoding a putative lytic enzyme homologous to a *N*-acetylmuramyl-L-alanine amidase (Fujimura and Murakami, 1997). But other mutations have been identified to provide the same type of phenotype, like mutations in *mecI* or in its promoter (Kondo et al., 2001). More recently, the comparison of the whole genome of strains selected from high and low level resistant subpopulations identified, in highly resistant strains, two additional mutations in *relA*, which is involved in the synthesis of (p)ppGppas, an effector of the stringent stress response to many environmental and genetic changes (Mwangi et al., 2013).

Ryffel et al. (1990) and Berger-Bachi and Rohrer (2002) first hypothesized that the heterogeneous-to-homogeneous conversion of methicillin resistance results from a spontaneous chromosomal mutation that is not linked to *mecA*. Kondo et al. (2001) showed by *in vitro trans*-complementation studies that *hmrA* and *hmrB*, which encode a putative aminohydrolase and an acyl carrier protein, respectively, were responsible for the conversion of the heterogenous profile (eagle type) of the N315 pre-MRSA strain into a uniformly highly resistant MRSA strain. Almost 20 years later a study wherein the whole genome of hetero-MRSA strain (N315) and its derivative homogeneously resistant strain selected by imipenem exposure, were compared confirmed that they differed in a single non-synonymous mutation in *rpoB*, encoding the RNA polymerase β subunit (Aiba et al., 2013). Furthermore more recently, WGS revealed that tandem amplification of the SCC*mec* near its integration site was another alternative mechanism driving the heterogenous-to-homogeneous conversion (Gallagher et al., 2017).

## Concluding Remarks

The development of *mecA*-mediated resistance to β-lactams was induced by human use of β-lactam antibiotics both to treat human infections and feed additives and involved several key genetic events: (1) the evolution of a native gene into a resistance determinant occurring at the native location; (2) the evolution of the SCC elements occurring at the *orfX* region; (3) integration of the *mec* complex and neighboring regions into a SCC element; (4) the adaptation of the host bacteria genetic background; (5) dissemination of SCC*mec* among staphylococci colonizing animals; (6) dissemination of SCC*mec* among staphylococci colonizing both animals and humans. Strikingly, most of the events that lead to β-lactam resistance development have occurred within the group of the most primitive animal-related *Staphylococcus* species isolated from production animals or human infection, suggesting it was a bacterial survival strategy against the human use of antimicrobials. The jump of SCC*mec* from animal to human-associated *Staphylococcus* species, like *S. aureus*, was a key event leading to several worldwide pandemics.

## Author Contributions

The author confirms being the sole contributor of this work and has approved it for publication.

## Conflict of Interest Statement

The author declares that the research was conducted in the absence of any commercial or financial relationships that could be construed as a potential conflict of interest.

## Footnotes

1 http://www.sccmec.org/Pages/SCC_TypesEN.html
